# Senescence-Associated Secretory Phenotype of Cardiovascular System Cells and Inflammaging: Perspectives of Peptide Regulation

**DOI:** 10.3390/cells12010106

**Published:** 2022-12-27

**Authors:** Vladimir Khavinson, Natalia Linkova, Anastasiia Dyatlova, Raisa Kantemirova, Kirill Kozlov

**Affiliations:** 1 Department of Biogerontology, Saint Petersburg Institute of Bioregulation and Gerontology, 197110 Saint Petersburg, Russia; 2 Group of Peptide Regulation of Aging, Pavlov Institute of Physiology of Russian Academy of Sciences, 199034 Saint Petersburg, Russia; 3 The Department of Therapy, Geriatrics and Anti-Age Medicine, Academy of Postgraduate Education under of FSBU FSCC of FMBA of Russia, 125371 Moscow, Russia; 4 Department of Therapy, Federal Scientific Center of Rehabilitation of the Disabled Named after G.A. Albrecht Ministry of Labor and Social Protection, 195067 Saint Petersburg, Russia; 5 Department of Hospital Therapy, Saint Petersburg State University, 199034 Saint Petersburg, Russia; 6 First Department and Clinic of Surgery, S. M. Kirov Military Medical Academy, 194044 Saint Petersburg, Russia

**Keywords:** SASP, inflammaging, peptides, cardiovascular pathology, aging

## Abstract

A senescence-associated secretory phenotype (SASP) and a mild inflammatory response characteristic of senescent cells (inflammaging) form the conditions for the development of cardiovascular diseases: atherosclerosis, coronary heart disease, and myocardial infarction. The purpose of the review is to analyze the pool of signaling molecules that form SASP and inflammaging in cells of the cardiovascular system and to search for targets for the action of vasoprotective peptides. The SASP of cells of the cardiovascular system is characterized by a change in the synthesis of anti-proliferative proteins (p16, p19, p21, p38, p53), cytokines characteristic of inflammaging (IL-1α,β, IL-4, IL-6, IL-8, IL-18, TNFα, TGFβ1, NF-κB, MCP), matrix metalloproteinases, adhesion molecules, and sirtuins. It has been established that peptides are physiological regulators of body functions. Vasoprotective polypeptides (liraglutide, atrial natriuretic peptide, mimetics of relaxin, Ucn1, and adropin), KED tripeptide, and AEDR tetrapeptide regulate the synthesis of molecules involved in inflammaging and SASP-forming cells of the cardiovascular system. This indicates the prospects for the development of drugs based on peptides for the treatment of age-associated cardiovascular pathology.

## 1. Introduction

**Figure 1:**
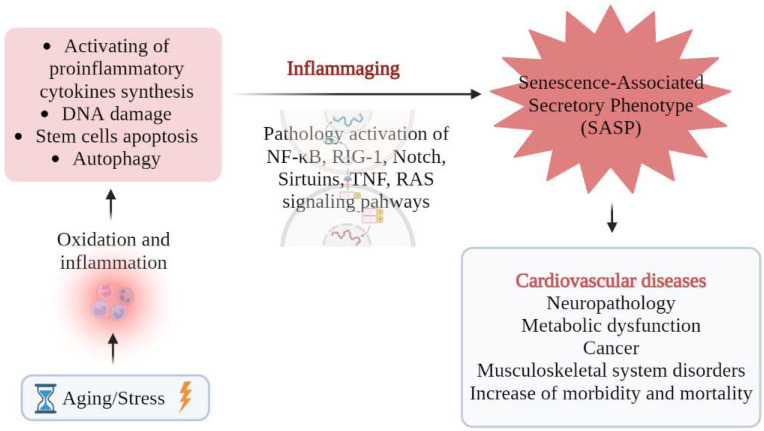
SASP, inflammaging and age-associated diseases.

Cellular aging is considered as one of the regulatory mechanisms that can prevent the uncontrolled proliferation of damaged cells. Cellular aging is also characteristic of differentiated cells exposed to damaging factors (prooxidant molecules, radiation, and chemotherapy) [1]. A large number of senescent cells are accumulated in tissues both during natural aging and in age-associated diseases, including cardiovascular diseases (CVD), Diabetes Mellitus type 2 (DM2), musculoskeletal system disorders, various types of tumors, and neurodegenerative disorders (Figure 1). Selective removal of senescent cells leads to an increase in the lifespan of animals [2]. 

Senescent cells are reported to contribute to the development of age-associated diseases, although the mechanisms of this process are not fully understood. It is assumed that this process occurs through the formation of a senescence-associated secretory phenotype (SASP). SASP is characterized by the secretion of signaling molecules, including proinflammatory mediators and extracellular matrix (ECM) degrading enzymes. SASP contributes to the development of a chronic, systemic, and mild inflammation called inflammaging. Inflammaging is one of the main risk factors for the development of age-associated diseases, including CVD [3].

The development of peptide geroprotectors that contribute to the achievement of the species lifespan limit and the preservation of basic physiological functions is one of the most urgent tasks of biogerontology and molecular medicine. In studies focused on this problem, considerable attention is paid to the role of short peptides in the prevention of accelerated aging [4].

Peptide regulation of homeostasis occupies an important place in the complex chain of physiological processes that lead to aging of cells, tissues, organs, and the organism as a whole. Polypeptide complexes isolated from various organs and tissues, as well as their biologically active components (di-, tri-, tetrapeptides), have been established to possess a pronounced tissue-specific activity both in cell culture and in experimental models in young and old animals [4,5]. The main advantages of short peptides over polypeptides include the lack of species-specificity and immunogenicity. These characteristics bring regulatory short peptides closer to peptide hormones [6,7,8].

Studies on short peptides at three levels (cells, tissues, and organism) have shown their safety and effectiveness. The use of peptide preparations in humans contributed to the restoration of basic physiological functions and a significant decrease in mortality in various age groups during a follow-up period of 6–12 years [6]. In this regard, further study of the mechanisms of the geroprotective action of peptides opens up new prospects for the development of the concept of peptide regulation of aging, as well as for the prevention of accelerated aging and age-related pathology, and prolongation of active longevity period. 

CVD, in particular coronary heart disease (CHD) and its acute form, myocardial infarction (MI), have shown the highest mortality rates over the past decade. One of the prerequisites for the development of coronary heart disease and myocardial infarction is atherosclerosis, a disease characteristic of elderly and senile people and characterized, among other things, by inflammatory damage to the vascular endothelium during the formation of atherosclerotic plaques. SASP molecules are involved in the formation of chronic systemic inflammation, which plays an important role in the pathogenesis of atherosclerosis and concomitant CVDs.

The purpose of the review is to analyze the pool of signaling molecules that form SASP and inflammaging in the cardiovascular system (CVS) tissues’ cells, as well as to search for possible targets for vasoprotective peptides.

The review considers the mechanisms of the SASP formation in endotheliocytes, vascular smooth muscle cells (VSMC), cardiomyocytes, the connection of SASP to the cardiovascular system, and the concept of inflammaging. New possible targets for the pharmacotherapy of age-related CVS pathology have been proposed.

## 2. SASP: Role in the Pathogenesis of Age-Related Cardiovascular Pathology

Senescent cells influence the microenvironment through auto/paracrine mechanisms, secreting many different factors, including cytokines, chemokines, proteases, and growth factors. The combination of these molecules forms SASP, which determines various processes in the body associated with regeneration [9], tissue remodeling [10], inflammation [11], embryogenesis [12,13], and carcinogenesis [14]. To date, the classification of the accepted SASP components is as follows: soluble signaling factors, proteases, insoluble ECM proteins, and non-protein components. According to the molecular mechanism of action, there are receptor-interacting factors (soluble signal molecules: cytokines, chemokines, and growth factors), direct-acting factors (matrix and serine proteases, non-protein components, reactive oxygen species (ROS), and nitric oxide), regulatory factors (tissue matrix metalloprotease inhibitors, plasminogen activator inhibitors, etc.). Apart from that, the extracellular miRNA-associated vesicles, which can both initiate and suppress cellular senescence depending on the types of miRNAs contained, have recently begun to be considered as SASP factors [15].

SASP is a dynamic process that can be divided into several phases. The first phase starts immediately after the DNA damage occurs, and lasts around 36 hours. The next phase of "early" SASP lasts several days and is characterized by increased cytokine synthesis. Then, within 4–10 days, the secretion of most SASP factors increases through autocrine exposure, which leads to the “mature” phase of SASP [16]. SASP regulation occurs at both transcriptional and post-transcriptional levels. The NF-κB factor plays the key role in the expression regulation of genes that are the main components of SASP [17]. 

Senescent cells accumulation and long-term SASP secretion may result in disrupted tissue function, accelerated aging rate, and the development of age-associated pathologies, including CVS. Increased MMPs expression by senescent cells plays an important role in the CHD progression. Pro-inflammatory cytokines secreted by senescent VSMCs contribute to the development of atherosclerosis [18]. Among the SASP components of CVS, SASPs of endothelial cells, VSMCs, and cardiomyocytes can be distinguished. 

### 2.1. SASP of Endothelial Cells

Compared to young endothelial cells (ECs), senescent ECs manifest a number of structural and functional changes [19]. These cells are characterized by the synthesis of pro-inflammatory and prothrombotic factors that contribute to the development of age-associated diseases [20]. Moreover, compared to young ECs, senescent ECs have a specific metabolic activity pattern, expressed in a decrease in glycolysis and an increase in glutaminolysis. [21]. 

The main factors causing accelerated aging and formation of SASP in endotheliocytes include DNA damage, mitochondrial dysfunction, blood flow disorders, oxidative stress, telomere shortening, and radiation [22].

Senescent ECs produce various SASP components depending on the type, strength, and duration of exposure to a stressor and microenvironment cells. Replicatively aging HUVECs produce IL-8, IL-6, PAI-1, and MCP-1 [23,24]. HUVECs with stress-induced aging type produce large amounts of the CXCL11 chemokine [25]. If HUVECs’ aging is mechanically induced by the simulation of blood flow disturbance, an increased production of ROS is observed, which is not characteristic of HUVECs during replicative aging [26]. The expression of some molecules, such as IL-1α, is characteristic of both replicative and stress-induced aging of HUVECs [27]. Exposure to growth factors, such as TGF-α, leads to the formation of SASP in HUVECs, which is dominated by the secretion of adhesion molecules (E-selectin and ICAM1), cytokines (IL-6 and IL-8), PAI-1, and IGFBP-5 [28]. SASP factors being secreted with doxorubicin-induced senescence of HMEC-1 ECs cause in vitro platelet activation, and, apparently, contribute to thrombosis and development of cardiovascular pathology [29]. An increase in MMP-9 level in the endothelium leads to inflammation. Thereby, a high level of MMP-9 has been identified as a sign of endotheliocyte inflammaging [30]. Similar to MMPs, MCP-1 (a member of the C-C chemokine subfamily, which mediates its effects through the CCR-2 receptor) also contributes to endotheliocyte senescence [31]. There is a gender dependence of the SASP expression profile, which has been shown in experiments on mice. [32]. This was probably due to differences in the production of sex hormones and the expression of their receptors [33]. 

For several years, attempts have been made to identify the representative markers of the EC aging, which would make it possible to identify aging ECs at different stages of the life cycle. Transcriptome analysis revealed differences in the expression of 68 RNA molecules in fibroblasts and senescent ECs [34]. 

The process of cellular aging is mainly controlled by two effector pathways: p53-p21 and p16-retinoblastoma protein (pRB) [35]. This being said, the p53/p21 pathway seems to play an essential role in the initiation of aging, while the p16-pRb pathway is instrumental to maintaining aging. Activation of the p16-pRb pathway draws a line between the two phases of aging: an early reversible phase dominated by p53 activity and an irreversible phase induced by the p16-Rb pathway [36].

Antiproliferative protein p53 acts as a tumor suppressor. As known, p53 is activated upon the accumulation of DNA damage, which results in the arrest of the cell cycle and DNA replication, and initiation of apoptosis in case of a strong stress signal. In the absence of cellular stress, p53 is deactivated by the action of MDM2 and MDM4 proteins, which promote polyubiquitination, nuclear export, and proteasomal degradation of p53. Under stress, phosphorylation of p53 amino terminus prevents MDM2 binding, which leads to the stabilization of the p53 protein and its acetylation. On the other hand, SIRT1 histone deacetylase is another inhibitor of the p53 activity, causing its deacetylation. p53 is crucial for the maintenance of endothelial homeostasis. p53 was found to disrupt endothelium-dependent vasodilation, which is necessary for maintaining normal blood circulation [37]. It has been shown that p53 mediates angiotensin II (Ang II)-induced impairment of vasodilation [38]. p53 inactivates eNOS by inhibiting its phosphorylation at Ser1177 [39]. eNOS inhibits apoptosis of ECs and VSMCs, while a decrease in the bioavailability of eNOS is the primary cause of endothelial dysfunction [33]. Meox transcription factor upregulates p53 expression in ECs, reduces the content of eNOS, and provokes endothelial dysfunction [40]. 

The p21 protein controls cell cycle progression and apoptosis in mature ECs and regulates the size and cyclicity of the pool of hematopoietic precursor cells [41]. The p53-p21 pathway appears to be more sensitive to the induction of EC senescence than the p16-Rb pathway. Expression knockdown of p53 (but not p16) inhibits EC senescence induced by various stimuli. For instance, the induction of aging by mechanical stimuli that simulate blood flow disorders leads to endothelium aging along the p53–p21 pathway [42]. Oxidized low-density lipoproteins of diabetic patients contribute to the aging of EC precursors, obtained from healthy donors, via the p53-mediated pathway [43]. In general, the p53-mediated endothelial aging results in the endothelial microenvironment dysfunction and further disruption of the endothelial barriers balance. The involvement of p53 in the pathogenesis of atherosclerosis and vascular remodeling in pulmonary hypertension has been shown [44]. 

Exposure to non-enzymatically glycosylated collagen resulted in senescence induction of the HUVECs line at early passages. At the same time, an increased expression of mRNA p53, p21, and p16 was observed. Suppression of p53 expression led to the inhibition of aging caused by overexpression of p16; suppression of p21 expression produced no such effect. On the other hand, Rb downregulation abolished senescence initiated by overexpression of p16 or p21 [45]. 

Thus, SASP of endothelial cells differs depending on their aging type: replicative or stress-induced. In addition, the type, strength, and duration of exposure to a stress factor are of great importance for the stress-induced type. Some molecules, such as IL-1α, are expressed in both replicative and stress-induced aging. In addition, the expression profile of SASP appears to be gender dependent. Signaling pathways of antiproliferative proteins p53-p21 and p16-pRb mediate EC aging, induced by various stimuli, to different extents.

### 2.2. SASP of Vascular Smooth Muscle Cells

Vascular smooth muscle cells (VSMCs) are an integral structural component of the arteries. They play an important role in the functional state of the vessels. The senescence of VSMC can be caused by many factors such as angiotensin II (Ang II), oxidative stress, inflammation, DNA damage, and exposure to low-molecular compounds. These inducers are able to genetically and epigenetically regulate VSMC senescence and SASP formation, which contributes to chronic vascular inflammation, arterial dysfunction, and the development of age-associated CVS diseases. 

Aging VSMCs have a low ability for mitotic division and are characterized by the changes in cell signaling, for example, high activity of SA-β Gal; increased levels of antiproliferative proteins p16, p38, p53, p21; and H2A.X histone phosphorylation [46]. In addition, there are specific characteristics of VSMC senescence: changes in the VSMC response to vasoconstrictors and vasodilators, a changes in the VSMC phenotype (from contractile to synthetic), changes in the specific VSMC signaling pathways (protein kinase G-1 (PKG-1) and potential-dependent and Ca2^+^-activated K^+^ (BKCa) channels), as well as violations of the relationship between VSMCs and extracellular matrix [47]. The inflammaging-induced SASP profile of VSMCs is characterized by the production of pro-inflammatory cytokines, including IL-1α, -1β, -6, -8, -18, and TNF-α [48]. By secreting cytokines, IL-1α in particular, senescent aortic VSMCs promote the transition of neighboring cells into pro-adhesive and inflammatory state [49]. Chronic inflammation is exacerbated by ROS production and a decrease in the antioxidant capacity of VSMCs [50]. This leads to the progression of atherosclerosis and other age-associated CVDs.

The renin-angiotensin-aldosterone system (RAAS) is one of the most important signaling pathways that activate VSMC senescence. Ang II affects the factors that ensure the migration of VSMCs, including monocyte chemotactic protein-1 (MCP-1), calpain-1 and MMPs [51]. Ang II is the activator of NF-κB, TGF-β, and matrix metalloproteinase system (MMPs), which makes it a key factor in the development of arterial inflammation and cellular senescence [52]. 

The following three signaling pathways are instrumental for the RAAS-mediated VSMC senescence: MMPs, MCP-1, and TGF-β1. MMPs have been shown to have multiple effects on VSMCs, including proliferation (MMP-9), migration and modulation of the ECM adhesion (MMP-1,-2,-9), and relaxation (MMP-2,-9) [53]. In addition to these effects, activation of MMP-2 in VSMCs under the influence of Ang II induces age-related changes in the vascular wall: calcification and fibrosis [54]. 

TGF-β1 is cleaved from pro-TGF-β1. TGF-β1 release is observed during inflammation and/or activation of MMP-2/9 [55]. Ang II can also activate TGF-β1 [56]. TGF-β1 increases the expression of collagenase and stromelysin and reduces the expression of the metalloproteinase-1 (TIMP-1) tissue inhibitor, which leads to in vitro cell senescence [57]. Overexpression of TGF-β1 is associated with increased arterial stiffness [58]. TGF-β1 in SASP acts as a mediator in the paracrine mechanism of cellular senescence; it is able to initiate the aging of other cells and enhance SASP of VSMCs [59]. 

Ang II accelerates atherosclerosis development by inducing premature VSMC senescence via the p53-p21-dependent pathway [60]. Senescent VSMCs also secrete proteases that destroy ECM, contributing to the destabilization of atherosclerotic plaques. Collagen secretion in senescent VSMCs is reduced compared to normal VSMCs [61]. 

Calcification is one of the signs of VSMC cellular senescence. Senescent VSMCs have been shown to overexpress genes and proteins (RUNX-2, alkaline phosphatase (ALP), type I collagen and BMP-2) associated with osteoblasts, which led to their partial transdifferentiation. It has been suggested that senescent VSMCs contribute to the CVS dysfunction by inducing vascular calcification [62]. 

The NF-κB transcription factor plays an integral role in the expression of many inflammation-associated genes [63]. NF-κB is believed to promote the development of CVD in arterial tissues by enhancing the proinflammatory and prooxidant gene transcription [64]. It has been established that NF-κB activity is higher in the cardiac tissues of old rodents compared to young ones [26,65]. During aging in vitro, VSMCs are characterized by overexpression of NF-κB [66]. Transcription factors NF-κB and C/EBPβ are activated in cell chromatin during cell aging and determine SASP profile by regulating the transcription of IL-8 or IL-6. In turn, IL-6 and IL-8 enhance the activity of C/EBPβ and NF-κB through autocrine feed-forward connection and activate the SASP signaling pathway [67]. C/EBPβ has been shown to regulate other SASP factors, including IL-1β, GROα, and NAP2 [52]. NF-κB upregulates many genes encoding pro-inflammatory cytokines and, thus, acts as the primary regulatory factor of SASP [68]. 

As already mentioned, extracellular microvesicles containing microRNAs can also induce cellular senescence. Microvesicles with miRNA or long non-coding RNA (lncRNA) induce VSMC senescence in vitro [69]. Increased expressions of miR-34a, as well as reduced expression of SIRT1, were observed during replicative aging of human aortic VSMCs. Overexpression of miR-34a in proliferating human aortic SMCs caused cell cycle arrest along with elevated levels of the p21 protein and signs of cellular senescence. In addition, miR-34a ectopic expression induced the synthesis of pro-inflammatory SASP molecules. miR-34a overexpression led to a decrease in SIRT1 synthesis. Raising SIRT1 level to normal values slowed the miR-34a-dependent aging of human aortic SMCs and did not invoke the activation of SASP. Thus, an increase in miR-34a expression contributes to VSMC aging and inflammation through the suppression of SIRT1 and induction of SASP factors, which can result in pathological changes in the aorta [70]. 

Studies of prelamin A as a marker of VSMC senescence are of interest. The cell nucleus homeostasis changes with aging. Nuclear plate defects are associated with several diseases, including Hutchinson-Gilford progeria syndrome (HGPS) [71]. HGPS is a severe genetic disease caused by point mutation that disrupts nuclear lamin A processing, resulting in the formation of mutant prelamin A (progerin) [72]. HGPS patients develop early arteriosclerosis, characterized by calcification and VSMC depletion, as well as pronounced adventitial fibrosis. This disease leads to death in adolescence, mainly due to MI or stroke. Overexpression of prelamin A accelerates VSMC aging by inducing DNA damage, which results in mitosis disruption, genome instability, and premature aging [73]. 

Thus, the SASP of VSMCs is characterized by increased synthesis of antiproliferative proteins (p16, p21, p38, p53), pro-inflammatory cytokines characteristic of inflammaging (IL-1α,β, IL-6, IL-8, IL18, TNFα, TGFβ1, NF-κB), matrix metalloproteinases (MMP1, MMP2, MMP9), miR-34a, and decreased expression of SIRT1. 

### 2.3. SASP of Cardiomyocytes

Premature cardiomyocyte aging plays a crucial role in the heart senescence and development of cardiac failure. Aging of cardiomyocytes leads to cardiac hypertrophy, arrhythmias, and other types of cardiomyopathy[74]. Senescent cardiomyocytes possess several morphological and molecular features that can serve as markers and therapeutic targets. Such cells are characterized by a flattened shape and larger size [75], increased activity of SA-β-gal, and shortening of telomeres [76]. 

In senescent cardiomyocytes, DNA damage, endoplasmic reticulum stress, mitochondrial dysfunction, impaired contractile activity, hypertrophic growth and expression of SASP molecules are observed. Cardiomyocyte aging is regulated by ECs, fibroblasts, and immune cells in their microenvironment. Senescent cardiomyocytes change their phenotypes and subsequently affect the cells of the microenvironment, promoting pathological remodeling of the cardiac tissue [77]. Dysfunctional ECs secrete pro-inflammatory factors (TGF-β, IL-6, and IL-33) and Ang II, modulating cardiomyocyte aging. Extracellular microvesicles or exosomes produced by ECs, which contain Mst1 kinase and miRNAs, also regulate cardiomyocyte function. Fibroblasts modulate cardiomyocyte aging through paracrine signaling and ECM remodeling by expressing MMPs, integrins, and fibronectin. Pro-inflammatory factors, including IL-11, IL-33, and extracellular vesicles containing miR-21-3p, osteopontin, and EGFR, are secreted by fibroblasts and modulate cardiomyocyte aging. Immune cell signals can also activate cardiomyocyte aging. Conversely, senescent dysfunctional cardiomyocytes express pro-inflammatory factors and chemokines, recruiting immune cells, and exacerbating the aging of fibroblasts and ECs in a paracrine way [77]. 

Chemotherapy, such as doxorubicin, can cause premature aging of cardiomyocytes with preferential SA-β-gal accumulation, cyclin-dependent kinase inhibitor (CDK-I) expression, and decreased cardiac troponin I phosphorylation and telomerase activity. This aging phenotype is associated with p53 acetylation [78]. Doxorubicin enhances mitochondrial DNA (mtDNA) damage in the myocardium of Wistar rats. Doxorubicin also increases the content of histamine in the left ventricle of the heart, which indicates the activation of mast cells during chemotherapy [74]. 

Metabolic factors such as carnitine palmitoyltransferase-1 (CPT1) and pyruvate dehydrogenase (PDH) are also the markers of senescent cardiomyocytes. CPT1 level in cardiac tissue of aged rats is significantly reduced. A decrease in CPT1 during aging can result in cardiovascular complications in various pathological processes [79,80]. CPT1 deficiency exacerbates cardiomyocyte senescence and hypertension-induced cardiac hypertrophy due to lipotoxicity [81]. 

Thus, cardiomyocyte senescence is modulated by their microenvironment: VSMCs, ECs, fibroblasts and immune cells secreting TGF-β, IL-6, IL-11, IL-33, Ang II and MMPs, which leads to shortening of telomeres, accumulation of SA-β-gal, ER stress, and hypertrophic growth of cardiomyocytes, resulting in SASP formation. 

**Figure 2:**
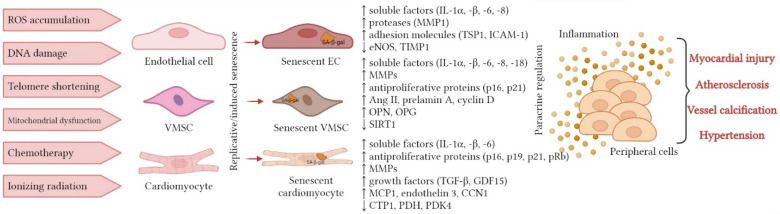
SASP factors involved in the formation of inflammaging in the local microenvironment of the cardiovascular system components.

Figure 2 shows the effects on the vascular wall components, which result in SASP formation, inflammaging, accelerated aging, and development of CVS diseases. Table 1 shows the cell type-specific molecular controller of SASP development. Negative stimuli such as ROS accumulation, DNA damage, telomere shortening, mitochondrial dysfunction, chemotherapy exposure, and ionizing radiation lead to the formation of SASP in endotheliocytes, VSMCs, and cardiomyocytes. As a result, changes in the molecular patterns of cytokine expression, matrix metalloproteases, antiproliferative proteins, growth factors, RAAS components, ECM proteins, etc. are observed. Through the paracrine mechanism, signal transmission from senescent to microenvironment cells occurs, resulting in the formation of chronic systemic inflammation–inflammaging. Inflammaging, in turn, through cytokine and chemokine signals, leads to an increase in the expression of SASP components. Prolonged exposure of the surrounding normal cells to SASP components leads to their accelerated aging and development of CVDs: atherosclerosis, coronary heart disease, hypertension, and MI.

## 3. Inflammaging: Role in the Pathogenesis of Age-Related Pathology of the Cardiovascular System

The term “inflammaging”, which was first used by Franceschi et al. in 2000, is associated with chronic subclinical inflammatory processes combined with biological aging [82]. The source of these inflammatory processes is still under debate, whereas SASP phenotype has been proposed as the underlying cause of inflammaging. SASP is characterized by the release of pro-inflammatory cytokines, increased activation of the NLRP3 inflammasome, changes in the nicotinic acetylcholine (ACh) receptor sensitivity, and abnormal NAD^+^ metabolism. NLRP3 is the main inflammatory sensor for intracellular DAMP molecules, which, together with damaged aggregate proteins released from destabilized lysosomes and damaged mitochondria, promote cellular stress and trigger NLRP3 activation. Upon activation, NLRP3 initiates an inflammatory response cascade by stimulating caspase-1, which activates proinflammatory cytokine precursors IL-1β, IL-1α, and IL-18 and their interaction with NF-κB. Although the initial activity of NLRP3 is low, the process of the inflammatory cascade initiation requires a complex oligomerization-priming phase that includes association with NF-κB [83].

Aging of endotheliocytes and VSMCs, formation of SASP and development of inflammaging are closely associated with numerous CVDs, including atherosclerosis [61,84], aortic aneurysm [85], and fibrous neointima formation [86]. Atherosclerosis occurs when the endothelium is damaged, which contributes to the accumulation of cholesterol-containing particles prone to oxidation in the arterial wall and causes a chronic inflammatory response. Activation of innate and adaptive immunity leads to the progression of atherogenesis, from early endothelial dysfunction to the development of acute thrombotic complications caused by plaque rupture or erosion. Monocytes that migrate into the arterial wall intima differentiate into macrophages and then transform into foam cells of the lipid-necrotic atheroma core. Cholesterol crystals and other DAMPs present in atherosclerotic lesions activate inflammasomes in macrophages, resulting in the release of IL-1β, IL-18, and other pro-inflammatory cytokines that are chemotactic for T- and B-cells. Advanced atherosclerosis is characterized by intense apoptosis and accumulation of senescent cells that maintain a pro-inflammatory status and lead to the formation of a necrotic core, which causes plaque rupture, thrombus formation, and acute vascular injury [87]. 

There is evidence that plaque-rich arteries contain SASP components, including MMPs and inflammatory factors. These SASP components are absent in normal adjacent blood vessels [88]. Senescent cells in blood vessels with SASP release various inflammatory cytokines (IL-6, IL-8) and growth factors (VEGF, PDGF, and chemokines) [89]. VSMCs derived from the thoracic aorta of older persons express higher levels of the platelet-derived growth factor receptor PDGFR-α and are resistant to apoptosis induced by nutrient deprivation or nitric oxide [90]. VSMCs derived from human atherosclerotic plaques have a lower proliferation level compared to the cells derived from normal parts of the arteries. Human VSMCs in plaques are characterized by higher expression of p16 and p21, pRB hypophosphorylation, increased SA-β-gal activity, and changes in cell morphology, compared to normal VSMCs [91]. Overexpression of the telomeric repeat-binding factor TRF2, which is specific for VSMCs, prevents aging and contributes to the stabilization of atherosclerotic plaques in apolipoprotein E (ApoE^-/-^) knockout mice [92]. 

VSMC senescence is associated with the enlargement of the necrotic core and plaque calcification in atherosclerosis [62]. With aging, VSMCs acquire a secretory osteoblastic phenotype and activate several osteogenic pathways via RUNX-2, BMP-2, ALP alkaline phosphatase, osteopontin (OPN), and osteoprotegerin (OPG), promoting plaque calcification. OPG, a soluble factor and key element of SASP, is a CVD risk factor. Plaque destabilization is facilitated by various MMPs, which are secreted as part of SASP of senescent VSMCs, monocytes, macrophages, and foam cells [93].

The NLRP3 inflammasome is considered as the main pathogenetic factor in the development of atherosclerosis, CHD, and ischemia-reperfusion injury of the heart. NLRP3 may become a new target for the prevention and treatment of CVD. Inflammasome is a macromolecular intracellular complex that provides a platform for stimulating the maturation of the pro-inflammatory cytokines IL-1β and IL-18. These cytokines are activated in infection, trauma or stress, and CVD. The NLRP3 inflammasome can be activated by PAMP and DAMP molecules and promote the secretion of IL-1β and IL-18 in CVD. The exact role of the NLRP3 inflammasome in the pathogenesis of hypertension, arrhythmias, and heart failure remains unclear. In the process of endotheliocyte stimulation by exogenous substances or endogenous mediators, oxidative stress, endoplasmic reticulum stress, and mitochondrial dysfunction occur, and the signaling pathway of the NLRP3 inflammasome activation is triggered. All the above leads to endothelial dysfunction [94]. Clinical trials have confirmed that IL-1β and its receptor antagonist can be used for the treatment of various CVDs; clinical trials have also proved that the drug glyburide plays a critical role in the treatment of CVDs by inhibiting the NLRP3 inflammasome [95]. 

Inflammaging is a complex systemic process that results from the interaction of several factors. SASP is considered as one of the inflammaging development factors. Changes in the secretory phenotype of endothelial cells, cardiomyocytes, and VSMCs, caused by various exogenous and endogenous factors, occur in the process of natural and induced aging, resulting in the SASP profile formation. These factors include DNA damage, mitochondrial dysfunction, telomere shortening, and oxidative stress. An increase in the number of cells with SASP leads to the development of chronic, systemic, and mild inflammation–inflammaging, which is one of the main risk factors for the development of age-associated CVDs [96]. 

## 4. SASP and Inflammaging Molecules as Possible Targets for Pharmacotherapy of Cardiovascular Pathology

Among the methods of CVD therapy, several approaches can be distinguished: senolytic strategy (selective elimination of senescent cells) and the senomorphic approach (SASP inhibition without the activation of senescent cells apoptosis). Senolytics are a new class of drugs that are able to distinguish between senescent and “young” cells to selectively induce apoptosis of the former. This hypothesis was first tested on transgenic INK-ATTAC mice, wherein the “death gene” was built into the p16Ink4a locus. In this case, apoptosis was activated by introducing the AP20187 molecule [3]. The removal of senescent cells contributed to the functional activity of organs and tissues and life expectancy at the organismal level. Therefore, considerable efforts have been directed to the identification of compounds that could selectively affect senescent cells [97]. Several senolytic candidates that target proteins of the BCL-2 family have emerged. These include ABT263 [98], A1331852, A1155463, and UX1325 [99]. The tyrosine kinase inhibitor dasatinib has several targets, including ephrin B2, which activates anti-apoptotic signals in senescent cells. Flavonoids such as quercetin and fisetin are naturally occurring compounds present in fruits that possess multiple effects and are senolytic in certain cell types. These effects include inhibition of PI3 kinase and plasminogen activator inhibitor-1 PAI-1. Due to the large number of anti-apoptotic pathways, some cell types require a combination of senolytics. The cardioprotective drug digoxin also possesses senolytic properties. Digoxin is a cardiac glycoside that enhances the exchange activity of Na^+^ and H^+^ ions, thus normalizing the cell pH. Senescent cells are more susceptible to changes in pH; thus, when this substance is used, a senolytic effect occurs [100]. 

Because SASP is a major contributor to aging-mediated tissue damage, treatments that reduce or modify the synthesis of these molecules are being explored. These treatments are known as senomorphic therapies. They have the potential to provide a significant proportion of the senolytic therapy benefits with a reduced toxicity profile. However, senomorphic therapy may be ineffective in intermittent regimens [101]. 

Despite their relative novelty, senolytic compounds are already undergoing clinical trials in malignant and benign neoplasms. In addition to malignant tumors, the BCL-2 family inhibitor, UX1325, is being tested in diabetic macular edema. Research into the effects of fisetin, dasatinib, and quercetin on skeletal muscle aging is currently ongoing. Their efficacy in osteoarthritis and Alzheimer’s disease is being evaluated. However, clinical trials of the senolytic therapy effectiveness in CVDs have not been conducted yet. The main difference between the treatment of CVDs compared to malignant diseases is the limited tolerance of toxicity and side effects. This narrows the range of applicability of senolytic therapy in atherosclerosis, but does not eliminate the need in search for effective substances of this group in vivo and in vitro CVD models.

Studies on senolytic therapy after myocardial infarction have been conducted. BCL-2 inhibitor ABT263 was administered to mice on the fourth day after acute MI simulation. ABT263 improved myocardial remodeling, left ventricular function and reduced animal mortality. In this case, SASP synthesis by heart muscle cells was reduced due to a decrease in profibrotic and pro-inflammatory signaling [102]. Preventive administration of ABT263 prior to MI modeling in mice promoted faster recovery of cardiac function and increased survival in animals after acute MI [103]. 

Selective apoptosis of P16INK4A^+^ senescent cells with the participation of senolytics in models of accelerated aging in INKATTAC and P16-3MR mice extend the period of active longevity, restore vascular functions, and stabilize atherosclerotic plaques [3,89,104]. Senescent endotheliocytes not only acquire SASP, but are also characterized by an increased ROS synthesis. Pharmacological Nrf2 activators, such as resveratrol, can attenuate ROS-induced DNA damage and inhibit ROS-induced senescence of CVS cells [105]. Phosphate binders (lanthanum carbonate and calcium carbonate) have been shown to prevent phosphate-induced VSMC aging and vascular calcification, while SIRT1 inhibits hyperphosphatemia-induced VSMC calcification [106]. Research aimed at eliminating senescent cells suggests that this approach will as well eliminate inflammaging [107].

Several of the currently available drugs and compounds, including antioxidants, statins, angiotensin-converting enzyme inhibitors, and Ang II receptor blockers, are capable of preventing premature CVS cell senescence and atherosclerosis by reducing ROS synthesis and oxidative DNA damage [48,61].

Senomorphic substances suppress the pro-inflammatory properties of senescent cells and slow down SASP formation by regulating many biochemical pathways involving m-TOR, p38-MAPK, NF-κB, JAK/STAT, ROCK, and glucocorticoid receptors. SASP components vary depending on the type of senescent cells, the causes of aging, and the endocrine profile of the organism. Therefore, hormones and several substances, including rapamycin, metformin, JAK1/JAK2 inhibitors, and glucocorticoids, can inhibit SASP [48]. For instance, rapamycin can reduce SASP synthesis in senescent fibroblasts by inhibiting m-TOR dependent translation of IL-1α mRNA and JAK/STAT3 pathway. In addition to anti-inflammatory properties, rapamycin inactivates the m-TOR pathway and exhibits geroprotective properties [108]. Many NF-κB inhibitors, including BMS-06 and vinpocetine, have been shown to slow the progression of atherosclerosis in mice. Experimental data indicate that inhibitors of the p38-MAPK pathway reduce SASP synthesis and in vitro senescent paracrine signaling [109]. Statins are effective in inhibiting the development of inflammaging in the arterial wall and the progression of atherosclerosis. Statins have been shown to slow the aging of endotheliocytes and T cells via p38-mediated SASP inhibition and cell cycle regulation [110]. 

Statins are used in older patients to normalize blood cholesterol levels and prevent atherosclerosis-related complications. Statins prevent cholesterol synthesis by inhibiting β-hydroxy-β-methylglutaryl-coenzyme A (HMG-CoA) reductase, the rate-limiting enzyme of the mevalonate pathway. Statins reduce the expression of “aging molecules” in endothelial precursor cells, which depends on the level of farnesyl pyrophosphate and geranylgeranyl pyrophosphate. Statins also prevent the formation of pro-inflammatory SASP in senescent fibroblasts [111]. Statins inhibit HMG-CoA reductase and reduce the synthesis of farnesyl-CoA and geranylgeranyl-CoA, which are necessary for the activation of Ras and Rac proteins that regulate DNA repair, apoptosis, aging, and are involved in the formation of SASP. However, statins can also act as radiosensitizers, increasing the activity of cancer cells in response to radiation [112]. There is evidence of a variety of effects of statins in relation to different signaling pathways involved in the development of atherosclerosis and senescence of CVS cells. For example, the activation of p53 during endothelial aging limits the pathways for the synthesis of mevalonates and cholesterol [113]. Thus, activation of p53 can slow down the development of SASP in endothelial cells [114]. It is likely that the relationship between aging, cholesterol, and atherosclerosis is more complex than previously thought.

Stress-induced DNA damage activated SASP formation through the formation of cytoplasmic chromatin. Interestingly, metformin and rapamycin-induced autophagy activation reduced the amount of cytoplasmic chromatin and inhibited the activation of the cGAS-STING-NF-κB cascade and cell senescence. These effects of autophagy activating factors explain why autophagic lysosomal function contributes to the removal of cytoplasmic chromatin and SASP inhibition. This confirms the role of the bafilomycin A1 lysosomal inhibitor in blocking the autophagy-mediated clearance of cytoplasmic chromatin and inhibiting cell senescence [115]. In addition, metformin has been shown to reduce the synthesis of pro-inflammatory cytokines by T-helpers via enhancing T cell autophagy. Metformin has also been shown to improve the biological function of mitochondria [116]. 

Thus, several groups of senolytics and senomorphic substances effective in CVP (cardio-vascular pathology) models can be distinguished: BCL-2 inhibitors (ABT263, UX1325, A1331852, and A1155463), regulators of Na^+^ and K^+^ ion exchange (digoxin), antioxidant drugs (rapamycin and metformin), immunosuppressants (rapamycin), and statins. 

## 5. Peptides: Prospects for the Regulation of the Cardiovascular System Functions during the Formation of SASP and Inflammaging

Peptides belong to one of the classes of effective drugs and geroprotectors with practically no side effects. Many peptides have a physiological mechanism of action and, according to their biological activity, can be classified as senomorphic substances. Short peptides consisting of two to seven amino acid residues can penetrate into the cytoplasm, nucleus, and nucleolus of the cell and interact with the nucleosome, histone proteins, and DNA [117,118,119].

Peptides can regulate DNA methylation status [120], which is an epigenetic mechanism of gene activation or repression in normal, aging, and pathological conditions. This raises the prospects for the development of effective and safe immunoregulatory, neuroprotective, antimicrobial, antiviral, and other drugs based on short peptides. In addition, the prospects for the use of short peptides as vasoprotective and cardioprotective agents were described [121,122,123].

In some cases, the development of synthetic drugs is based on physiologically significant peptides, such as GLP-1—glucagon-like peptide 1. GLP-1 is a peptide of about 30 amino acid residues, a product of tissue-specific post-translational processing of the proglucagon peptide. It is produced and secreted by intestinal enteroendocrine L-cells and some neurons in the solitary tract nuclei of the brain stem when food is consumed. The original GLP (1-37) is subject to amidation and proteolytic cleavage, resulting in the formation of two shortened and equipotent biologically active forms: amide-GLP-1 (7-36) and GLP-1 (7-37). The secondary structure of the active GLP-1 peptide includes two α-helices from amino acid positions 13–20 and 24–35 separated by a linker site. GLP-1 receptor (GLP-1R) agonists are known to reduce the risk of CVD in diabetes mellitus type 2 [124]. The GLP-1R agonist is a GLP-1 analogue, the liraglutide peptide, wherein lysine is replaced by arginine at position 34, and a C-16 fatty acid chain is added via a glutamic acid spacer residue at position 26. The mechanism of the observed vasoprotective effect of GLP-1 and its analogues is not completely clear. Liraglutide administration in a non-diabetic mouse model of arterial hypertension normalized blood pressure and reduced the severity of cardiac hypertrophy, vascular fibrosis, endothelial dysfunction, oxidative stress, and inflammation. Thus, liraglutide peptide produces a vasoprotective effect by interacting with the GLP-1 receptor on endothelial cells [125]. In a mouse model of hypertension induced by continuous infusion of Ang II via an implanted osmotic pump, liraglutide at a dose of 400 μg/kg per day reduced blood pressure and blood sugar, and also inhibited collagen accumulation, AT1R expression, and ROS formation in the heart. In in vitro studies, liraglutide pretreatment inhibited Ang II-induced ROS production and collagen expression in cardiac fibroblasts. Thus, the mechanism of inhibition of myocardial fibrosis by liraglutide apparently consists of a decrease in ROS production and regulation of collagen expression [126].

A pilot clinical study showed that liraglutide could inhibit the pro-inflammatory NF-κB pathway by upregulating SIRT1 expression in patients with obesity and diabetes mellitus type 2, improving their metabolic profile. A decrease in the mRNA expression of TNF-α, IkB, TLR2, TLR4, and ceruloplasmin in the blood mononuclear cells of patients occurred after six weeks of liraglutide treatment. At the same time, a significant increase in SIRT1 mRNA expression was observed. Six weeks after the discontinuation of liraglutide treatment, the mRNA expression of TNF-α, IkB, TLR2, TLR4, NOD1, SIRT1, and ceruloplasmin in blood mononuclear cells did not return to the baseline. At the same time, IL-2 expression decreased compared to the original level. These data demonstrate the mechanisms of liraglutide’s anti-inflammatory action, involving the inhibition of NF-κB pathways and upregulation of SIRT1 expression, downregulation of pro-inflammatory factors including cytokines (TNF-α), extra- and intracellular receptors (TLR2, TLR4), and inflammatory markers such as ceruloplasmin. The long-term effects of liraglutide may be mediated by epigenetic regulation of the NF-κB pathway involving SIRT1 [127].

The natriuretic peptide (NP) family, the atrial natriuretic peptide (ANP) in particular, plays an important role in the regulation of blood pressure homeostasis and electrolyte balance [128]. ANP consists of 28 amino acid residues; it is synthesized, stored, and released by cardiomyocytes. The release of ANP occurs in response to atrial stretch and a number of other signals induced by hypervolemia. In addition to their effect on the CVS, ANPs have been described as anti-inflammatory regulators of macrophage function. They have been reported to inhibit the induction of iNOS, COX-2, and TNF-a inflammatory mediators [129]. It is claimed the anti-inflammatory effect of ANP results from the modulation of leukocyte adhesion to the inflamed endothelium [130]. 

Carperitide, a recombinant human ANP, is used as a therapeutic agent for acute cardiac failure. Carperitide has been used to treat refractory heart failure due to severe acute MI [131]. Carperitide infusion improved the postoperative condition of patients with abdominal aortic aneurysm, presumably reducing hypertension and renal dysfunction postoperatively [132]. However, a cohort study conducted in Japan in 76,924 patients with acute cardiac failure demonstrated that the use of carperitide during early inpatient treatment was associated with worse outcomes compared to the use of nitrates [133].

The urocortin 1 (Ucn1) peptide, consisting of 40 amino acid residues, is a member of the corticotropin-releasing factor/urotensin I family. It causes prolonged hypotension and coronary vasodilation. In mice deficient in apolipoprotein E (ApoE^-/-^), intraperitoneal administration of Ucn1 for four weeks suppressed the development of atherosclerotic lesions in the aorta. Ucn1 reduced the formation of foam cells induced by oxidized low density lipoproteins and contributed to the suppression of macrophage expression of CD36, acyl-CoA, and cholesterol acyltransferase 1, which bind fatty acids. Ucn1 suppressed the migration and proliferation of human VSMCs, but increased the activity of MMP2 and MMP9 in them [134].

Relaxin peptides have vasoprotective and antifibrotic effects. In rats with spontaneous hypertension, relaxin administration reduced endothelial dysfunction by increasing NO-dependent relaxation and decreasing endothelium-dependent contraction. Short-term relaxin treatment increased the expression of the mesenteric PGI2 receptor (IP), providing PGI2-IP-mediated vasorelaxation [135]. Compared to other peptide-based drugs, relaxin has several limitations, such as poor oral bioavailability, complexity, and high cost of synthesis.

Recombinant relaxin-2 (serelaxin) has shown a positive effect in acute cardiac failure, but its clinical use has been hampered by a short half-life and the need for intravenous administration. This necessitates the development of long-acting single-chain peptide mimetics of relaxin. Modifications to the relaxin B chain, such as introducing specific mutations and trimming the sequence to an optimal size, have led to the creation of potent structurally simplified peptide agonists of relaxin receptor (RXFP1). The introduction of suitable spacers and fatty acids has led to the identification of single chain lipidized peptide agonists of RXFP1 possessing subnanomolar activity, high subcutaneous bioavailability, increased half-life, and in vivo potency [136].

Adropin, a highly conserved peptide hormone of 76 amino acid residues, is secreted predominantly in the liver. Recently, adropin has been considered as a regulator of vascular endothelial function in CVD [137,138]. In endothelial cells, adropine activates signaling via the mitogen-activated protein kinase (MAPK) pathway or via the VEGFR2 receptor. In cardiomyocytes, adropin acts through the GPR19 receptor, regulating the expression of pyruvate dehydrogenase kinase 4 (PDK4) and increasing the activity of pyruvate dehydrogenase (PDH) via ERK1/2 kinase. However, adropine has not been introduced into conventional clinical practice yet, since the therapeutic delivery of peptide hormones is limited due to their pharmacokinetic properties [139].

Adropin concentration in the blood is negatively correlated with the content of homocysteine, hypersensitive C-reactive protein (hs-CRP), and cytokine levels in coronary heart disease and atherosclerosis. It is known that homocysteine leads to the dysfunction of vascular endothelium and smooth muscle cells. The level of homocysteine negatively correlates with the concentration of adropin in the blood of patients with coronary heart disease [140]. Adropin can downregulate the expression of TNF-α and IL-6 by regulating iNOS expression and exerting anti-inflammatory and anti-atherogenic effects [141]. Adropin suppresses the expression of CD36, a receptor that induces inflammatory responses through the activation of various ligands. For example, the interaction of CD36 with fibrillar β-amyloid (fAβ)/integrin can induce an inflammatory response by increasing the expression of pro-inflammatory cytokines and chemokines. The participation of CD36 in the pathogenesis of atherosclerosis has been shown [142,143]. The content of adropin in the blood negatively correlated with C-reactive protein acute inflammation marker [144]. 

The KED peptide (Lys-Glu-Asp) possesses vasoprotective properties; it is also effective in the treatment of atherosclerosis and other CVD in older people [121,122,145]. One of the proposed mechanisms of the biological activity of this tripeptide is the epigenetic regulation of genes encoding proteins of the antioxidant system and endothelial functional activity. The study of the KED effect on the expression of signaling molecules in normal, atherosclerotic, and restenotic endothelium in vitro demonstrated that KED normalized the expression of endothelin-1, which increases in atherosclerosis and restenosis. The KED peptide also contributed to the cell-to-cell cooperation by increasing the expression of connexin, the cell junction protein. It has been established that the geroprotective effect of the KED peptide occurred due to an increase in the expression of SIRT1, involved in the DNA repair [121]. The KED peptide activated the synthesis of proteins that are markers of the functional activity of the vascular endothelium (Ki67, Cx43, and VEGF) and reduced the expression of the antiproliferative p53 protein during replicative senescence of endotheliocytes. An increase in the expression of the Ki67 protein (proliferation protein) under the action of the KED peptide might be due to its specific interaction with the CATC site in the DNA minor groove [146,147].

The data on the effect of the KED peptide on the expression of Ki67, p53, and VEGF proteins of human and rat endotheliocyte cultures during their aging may play an important role in understanding the molecular mechanisms of the antiatherosclerotic effect of this peptide, which was revealed upon oral administration in older patients [140,143]. It is known that the violation of the integrity of the arterial intima endothelial lining can be caused by apoptotic death of endothelial cells. The reason underlying the development of endothelial apoptosis in atherosclerosis may be the induction of lipid peroxidation and the accumulation of active oxygen radicals. A study conducted on the coronary arteries of patients with atherosclerosis and coronary heart disease at various stages of atherogenesis showed an increase in the apoptotic index of endotheliocytes in coronary arteries affected by atherosclerosis, as compared to the undamaged segments of vessels in the control group. At the same time, intensive apoptotic processes in vascular endothelium were typical only for the early stages of the atherosclerotic process. Therefore, stimulation of endotheliocyte proliferation under the action of the KED peptide may indicate the ability of this tripeptide to prevent the development of atherosclerosis at its initial stage. Thus, one of the most important molecular aspects of the vasoprotective action of the KED peptide is the regulation of processes of cell renewal and reduction of the level of endothelial apoptosis. According to molecular modeling data, the KED peptide can epigenetically regulate the expression of genes whose products maintain the functional activity of vascular endotheliocytes during aging [7,146].

Tht AEDR (Ala-Glu-Asp-Arg) tetrapeptide was detected in the polypeptide complex of the heart by highly sensitive chromato-mass spectrometry. Administration of the AEDR peptide in experimental MI (ligation of the coronary artery in rats) led to a threefold decrease in mortality after the heart attack as compared to the control. The drug administration in animals also resulted in the decrease in necrotic zones and preservation of glycogen content in the myocardial tissue. AEDR exerted a protective effect on mitochondria and stimulated reparative processes, which improved the cardiomyocyte metabolism [123]. In cell culture of mouse embryonic fibroblasts, AEDR increased the synthesis of cytoskeletal proteins (actin, tubulin, and vimentin), by two to five times, and of nuclear proteins, lamins A and C) by two to three times [148]. Perhaps this is how the AEDR peptide enhances intracellular metabolism and promotes the induction of cell proliferation. These data allow us to consider the AEDR tetrapeptide as a biologically active substance capable of enhancing the synthesis of cyto- and karyoskeleton proteins, which can be applicable in the prevention and treatment of diseases associated with violations of the integrity of myocardial regions in various heart pathologies.

Thus, several main targets for pharmacotherapy with drugs based on short peptides can be identified. The NF-κB pathway and increased SIRT1 deacetylase activity are regulated by the GLP-1 analogue Liraglutide. ANP exerts an anti-inflammatory effect through the inhibition of inflammatory mediators iNOS, COX-2, and TNF-α. It is also used in clinical practice for the treatment of MI. Urocortin 1 and adropin regulate the formation of lipid metabolism products and prevent the development of atherosclerosis. Relaxin peptides exert a vasoprotective effect due to the NO-dependent relaxation and reduction of endothelium-dependent contraction. The KED tripeptide regulates the synthesis of the functional activity proteins of the vascular endothelium (Ki67, Cx43, and VEGF) and reduces the level of the antiproliferative protein p53 during the replicative aging of endotheliocytes. The AEDR tetrapeptide demonstrates cardioprotective properties in vivo, reducing the mortality after experimental myocardial infarction in rats. 

## 6. Conclusions

Replicative and accelerated aging of CVS components—VSMCs, endotheliocytes, cardiomyocytes—leads to SASP. The molecular patterns of SASP expression are characterized by the secretion of a wide range of cytokines, chemokines, proteases, adhesion molecules, and extracellular vesicles. SASP affects microenvironment cells (fibroblasts and immune cells) via paracrine mechanisms, stimulating the secretion of pro-inflammatory factors that aggravate the process. This leads to the development of a chronic, indolent, systemic inflammation–inflammaging, which is characteristic of the senescent organism. The uncontrolled spread of inflammation leads to the accumulation of damage in the structural components of the cardiovascular system and contributes to the development of hypertension, atherosclerosis, coronary artery disease, and myocardial infarction. Elimination of senescent cells with SASP leads to a slowdown in the rate of aging in animals. Therefore, the search for targets, the impact on which can reduce the formation of SASP and inflammaging, is an urgent task of modern biogerontology and molecular cardiology. 

Literature data analysis showed the presence of biologically active substances aimed at normalizing the synthesis of SASP molecules. Physiologically active peptides stand at the head of them (Table 2). In addition to peptide hormones such as urocortin, adropin, and GLP-1, short peptides KED, and AEDR (active components of the polypeptide complexes of blood vessels and the heart) are of interest. They can epigenetically regulate the expression of genes and the synthesis of proteins involved in aging and maintaining the functional activity of vascular and cardiac cells. These peptides have some protective effects in experimental models of age-associated CVD: infarction of the myocardium, atherosclerosis, blood pressure normalization. Due to their small size and low molecular weight, these peptides provoke no allergic reactions and have no side effects compared to other senolytics. Currently, the mechanism of the short peptides’ transportation into the cell is only being studied. One of the hypotheses suggests the involvement of POT and LAT peptide carriers in this process [117]. However, there is already evidence of the successful use of some short peptides in clinical practice. 

Thus, the continuation of studies on the vaso-, cardio-, and geroprotective properties of short peptides as regulators of the CVS functions during its aging under normal and pathological conditions, taking into account the concept of SASP and inflammaging, is an actual vector of development for molecular medicine and biogerontology.

## Figures and Tables

**Table 1:** Comparative cell type-specific molecular controller of SASP development.

Cell Type	Molecular Controller of SASP Development
Endothelial cells	eNOS, TIMP1, TSP1, ICAM1
Vascular smooth muscle cells	IL-18, Sirt1, OPN, OPG, AngII, Prelamin A, Cyclin D
Cardiomyocytes	TGFβ, GDF15, pRb, p19, MCP1, Endothelin3, CCN1, CTP1, PDH, PDK4

**Table 2:** Short peptides with vasoprotective and cardioprotective properties.

Peptide	Amino Acid Sequence	Vasoprotective and Cardioprotective Properties	References
Tetrapeptide	AEDR	Reduces acute myocardial infarction-related mortality in experimental animals; produces a protective effect on mitochondria; stimulates reparative processes in the myocardium in the experimental infarction; regulates the synthesis of cyto- and karyoskeleton proteins in fibroblasts.	[123,148]
Tripeptide	KED	Regulates endothelial synthesis of SIRT1, endothelin-1, connexin, Ki67, Cx43, VEGF, p53 in aging, atherosclerosis and restenosis; normalizes blood circulation in elderly atherosclerosis patients.	[7,121,122,145]
Liraglutide	HAEGTFTSDVSSYLEGQAAKEFIAWLVRGRG	Reduces blood pressure, inhibits fibrosis in in vivo CVD models, has anti-inflammatory properties in the blood mononuclear cells culture.	[126,127,149]
Atrial Natriuretic Peptide (ANP)	MSSFSTTTVSFLLLLAFQLLGQTRANPMYNAVSNADLMDFKNLLDHLEEKMPLEDEVVPPQVLSEPNEEAGAALSPLPEVPPWTGEVSPAQRDGGALGRGPWDSSDRSALLKSKLRALLTAPRSLRRSSCFGGRMDRIGAQSGLGCNSFRY	Induces vasodilation, natriuresis, diuresis and counteracts the renin-angiotensin-aldosterone system (RAAS).	[128,129,130,132]
Human urocortin 1 (Ucn1)	DNPSLSIDLTFHLLRTLLELARTQSQRERAEQNRIIFDSV	Inhibits the formation of foam cells; inhibits the synthesis of CD36, acyl-CoA, cholesterol acyltransferase 1 in macrophages, reduces the migration and proliferation of human GMSCs, increases the activity of MMP2 and MMP9 in GMSCs.	[134,150]
Mimetics of Relaxin	DSWMEEVIKLCGRELVRAQIAICGMSTWSLYSALANKCCHVGCTKRSLARFC *(Serelaxin)*	Enhances NO-dependent relaxation, leads to PGI2-IP-mediated vasorelaxation	[135,136]
Adropin	MGAAISQGALIAIVCNGLVGFLLLLLWVILCWACHSRSADVDSLSESSPNSSPGPCPEKAPPPQKPSHEGSYLLQP	Negatively correlates with homocysteine, highly sensitive C-reactive protein, CD36, TNF-a, IL-6; regulates the expression of PDK4 and PDH enzymes, which ensure the metabolism of fatty acids and are involved in the formation of SASP in cardiomyocytes.	[137,138,139,141,143,144]

## Data Availability

Not applicable.
